# Heterologous Expression of the *Leuconostoc* Bacteriocin Leucocin C in Probiotic Yeast *Saccharomyces boulardii*

**DOI:** 10.1007/s12602-020-09676-1

**Published:** 2020-06-21

**Authors:** Ran Li, Xing Wan, Timo M. Takala, Per E.J. Saris

**Affiliations:** Department of Microbiology, Faculty of Agriculture and Forestry, University of Helsinki, Viikinkaari 9, P.O. Box 56, 00014 Helsinki, Finland

**Keywords:** Probiotic yeast, *Saccharomyces boulardii*, Bacteriocin, Leucocin C, Heterologous expression

## Abstract

The yeast *Saccharomyces boulardii* is well known for its probiotic effects such as treating or preventing gastrointestinal diseases. Due to its ability to survive in stomach and intestine, *S. boulardii* could be applied as a vehicle for producing and delivering bioactive substances of interest to human gut. In this study, we cloned the gene *lecC* encoding the antilisterial peptide leucocin C from lactic acid bacterium *Leuconostoc carnosum* in *S. boulardii*. The constructed *S. boulardii* strain secreted a peptide, which had molecular weight corresponding to leucocin C in SDS-PAGE. The peptide band inhibited *Listeria monocytogenes* in gel overlay assay. Likewise, concentrated *S. boulardii* culture supernatant inhibited the growth of *L. monocytogenes*. The growth profile and acid tolerance of the leucocin C secreting *S. boulardii* were similar as those of the strain carrying the empty vector. We further demonstrated that the cells of the leucocin C producing *S. boulardii* efficiently killed *L. monocytogenes*, also without antibiotic selection pressure. These results showed that antilisterial activity could be added to the arsenal of probiotic activities of *S. boulardii*, demonstrating its potential as a carrier for therapeutics delivery.

**Electronic supplementary material:**

The online version of this article (10.1007/s12602-020-09676-1) contains supplementary material, which is available to authorized users.

## Introduction

The probiotic yeast *Saccharomyces boulardii*, originally isolated from litchi fruit by Henri Boulard in the 1920s, is a subtype of *Saccharomyces cerevisiae* [1]. *S. boulardii* is the only approved probiotic yeast in human nutrition [2] and has been granted with the GRAS (generally regarded as safe) status by the Food and Drug Administration (FDA) of USA [3]. The benefits of *S. boulardii* have been widely assessed. Controlled clinical trials have indicated that oral administration of *S. boulardii* can treat or prevent gastrointestinal diseases such as antibiotic-associated diarrhea [4], acute diarrhea in children [5, 6], and AIDS-associated diarrhea [7]. Additionally, some studies have revealed that *S. boulardii* can control inflammation in the inflammatory bowel disease (IBD) like Crohn’s disease and ulcerative colitis [8].

Despite the probiotic effects of *S. boulardii* are well understood, only a few studies have focused on expanding its probiotic properties by production of heterologous proteins, such as cytokines or antimicrobial proteins. In the previous work, Douradinha et al. [9] investigated factors concerning the genetic manipulation of *S. boulardii*, such as plasmids transformation and screening of positive strains. Regarding heterologous protein expression, researchers have produced mouse interleukin-10 (IL-10) [10, 11] and Microneme-2 proteins of chicken parasite *Eimeria tenella* [12] in *S. boulardii* using antibiotics as selection markers. In addition, green fluorescent protein (GFP) [13], human lysozyme [14], and ovalbumin (OVA) [15] have been secreted in auxotrophic mutant of *S. boulardii*. Thus, regardless of the limited number of genetic studies, it is possible to produce heterologous proteins in *S. boulardii*. As a carrier to deliver drugs or antimicrobial agents to gastrointestinal tracts for treating disorders or killing pathogens, *S. boulardii* is more suitable than *S. cerevisiae*, due to the better tolerance of *S. boulardii* to high temperature and low pH [1]. To the best of our knowledge, so far there are no studies reported about the production of antimicrobial proteins from bacteria in *S. boulardii*.

Bacteriocins are ribosomally synthesized antimicrobial proteins produced by bacteria. Studies have shown that a large amount of lactic acid bacteria (LAB) strains including *Lactobacillus*, *Lactococcus*, and *Leuconostoc* can secrete bacteriocins, of which many have been well characterized [16]. Bacteriocins generally comprise three classes [17]. Among the class II group, the subgroup class IIa (pediocin-like) bacteriocins have high killing activity against *Listeria monocytogenes* [18] by holing its cell membrane and causing internals release [19]. *L. monocytogenes* is a foodborne pathogen and widely distributed in the environment, including water [20], foods [21], and human feces [22]. In human, *L. monocytogenes* causes infection known as listeriosis, one of the most severe foodborne diseases [23]. In 2017, there were 786 listeriosis cases reported in the USA [24] and 2480 cases in EU [25].

In our previous work, we have successfully produced the class IIa bacteriocin leucocin C from *Leuconostoc carnosum* 4010 [26] in *Lactococcus lactis* NZ9000 [27]. Therefore, in this present study, we chose leucocin C as the target antimicrobial peptide. The main aim was to design a recombinant *S. boulardii* strain, which could produce antilisterial leucocin C besides its probiotic characteristics. Based on the results of antimicrobial tests, SDS-PAGE, gel overlay assay, and *L. monocytogenes* killing assay, active leucocin C was shown to be successfully secreted by *S. boulardii*. The benefits of *S. boulardii* could thus be enhanced with the combination of antimicrobial and probiotic effects.

## Materials and Methods

### Plasmids, Strains, and Media

All the plasmids and strains used in this study are listed in Table 1. *Escherichia coli* DH5α was grown in LB medium (1% tryptone, 0.5% yeast extract, 1% NaCl) at 37 °C, supplemented with kanamycin (50 μg/ml) for selecting the transformants. *Saccharomyces boulardii* CNCM I-745 was grown in YPD medium (1% yeast extract, 2% peptone, 2% glucose) at 37 °C, and 20 μg/ml blasticidin S (Thermo Fisher Scientific, Waltham, MA, USA) was used to maintain plasmids in yeasts [28]. *L. monocytogenes* was grown in BHI (brain heart infusion, Lab M, Lancashire, UK) agar or in broth at 37 °C.

**Table 1:** Plasmids and strains

Plasmids and strains	Description	Source
Plasmids		
pSF-Blast	pSF-TEF1-TPI1-Blast (OG539), *E. coli*-yeast shuttle vector	Oxford Genetics, Oxford, UK
pSF-Blast-*lecC*	pSF-TEF1-TPI1-Blast-*lecC*, plasmid inserted with leucocin C expression cassette	GenScript, Piscataway, NJ, USA
Strains		
*Escherichia coli* DH5α	Library Efficiency™ DH5α™ Competent Cells; intermediate host for preserving plasmids	Invitrogen, Carlsbad, CA, USA
Sb-wild type	*Saccharomyces boulardii* CNCM I-745, wild-type strain	Capsule PRECOSA, Biocodex, Espoo, Finland
Sb-vector	*S. boulardii* CNCM I-745 carrying pSF-TEF1-TPI1-Blast (OG539)	This study
Sb-LecC	*S. boulardii* CNCM I-745 carrying pSF-TEF1-TPI1-Blast-*lecC* for leucocin C expression	This study
*Listeria monocytogenes* WSLC 1018	Indicator strain, sensitive to leucocin C, ATCC 19118	Prof. Martin Loessner, ETH Zürich, Switzerland

### Plasmid and Transformants Construction

**Fig. 1:**
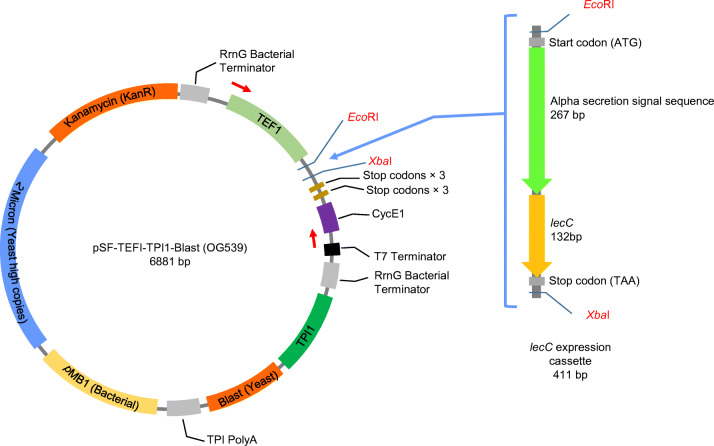
Construction of the plasmid pSF-Blast-*lecC*. The *lecC* expression cassette was cloned into *Eco*RI and *Xba*I sites in the vector pSF-TEF1-TPI1-Blast (OG539), resulting in the leucocin C secretion plasmid pSF-TEF1-TPI1-Blast-*lecC*. The red arrows on the plasmid map indicate the location where the plasmid specific primers would bind

For heterologous expression of leucocin C in *S. boulardii*, the plasmid pSF-TEF1-TPI1-Blast (pSF-Blast) was used as the expression vector (Fig. 1). This vector carries a blasticidin S resistance cassette (Blast), which is driven from the strong yeast constitutive triosephosphate isomerase (TPI1) gene promoter. Blast is the yeast selection marker, while the kanamycin-resistant gene (Kan^R^) is to select bacterial transformants. The plasmid also contains a constitutive promoter of yeast translation elongation factor 1 (*TEF1*), which is the strongest promoter known for the protein expression in *S. cerevisiae* [29, 30]. *Eco*RI and *Xba*I were selected as the restriction sites for the gene insertion.

The DNA fragment for the leucocin C secretion (Fig. 1) was synthesized by GenScript (Piscataway, NJ, USA). This fragment consists of the *S. cerevisiae* α-mating factor signal sequence to guide the extracellular secretion [31] and the *lecC* gene sequence [27]. Codon optimization was achieved by GenScript using OptimumGene™ algorithm to enhance the efficiency of gene expression. After optimization, the synthesized fragment was cloned into pSF-Blast plasmid at the *Eco*RI/*Xba*I sites. To increase the amount of plasmid for yeast transformation, the resulting plasmid pSF-Blast-*lecC* (pSF-TEF1-TPI1-Blast-*lecC*) was introduced into *E. coli* DH5α by electroporation [32]. Afterwards, the enriched pSF-Blast-*lecC* plasmid was isolated from *E. coli* with EZ-10 Spin Column Plasmid Mini-Preps Kit (BBI Life Sciences, Shanghai, China).

After that, 2.7 μg of recombinant plasmid was transferred into *S. boulardii* via electroporation [33]. Correct transformants were identified by PCR using the plasmid specific primers (F, 5′-CATATCACATAGGAAGCAACAG-3′, hybridizing before the *TEF1* promoter; R, 5′-CTACGATACCGATAGAGATGG-3′, hybridizing before T7 terminator), which resulted in PCR products of 1400 bp from pSF-Blast-*lecC* and 1050 bp from the plain vector.

### Secretion of Leucocin C

For the production of leucocin C, *S. boulardii* carrying pSF-Blast-*lecC* (Sb-LecC) was grown in 250 ml of broth at 37 °C for 36 h with shaking. *S. boulardii* wild type (Sb-wild type) and *S. boulardii* carrying empty vector pSF-TEF1-TPI1-Blast (Sb-vector) were used as negative controls. Cell-free supernatants were collected by centrifugation at 5000×*g* for 10 min at 4 °C and filtered through the 0.22-μm sterilized Millipore filter (Merck Millipore, Bedford, MA, USA). The leucocin C in the cell-free supernatant of Sb-LecC was precipitated with 40% ammonium sulfate and collected by centrifugation at 10,000×*g* for 30 min as previously described [26, 34]. The protein precipitation was dissolved in 500 μl of 20 mM PBS buffer (pH 7.2). The final concentrated samples were kept at − 20 °C for further tests. The concentrated supernatant from the leucocin C producing *L. lactis* NZ9000 constructed in our previous study [27] was used as a positive control.

### Antimicrobial Tests

An agar inhibition assay [35] was performed to roughly estimate the antimicrobial activity of recombinant *S. boulardii* cells. Yeast cells were grown in the YPD liquid culture overnight and washed with Ringer’s solution (Sigma-Aldrich, St. Louis, MO, USA) for two times. Ten microliters of the washed cells were added and let dry on the *Listeria* indicator plate. After overnight incubation, the inhibition halos were observed to determine the antimicrobial activity of yeast cells.

The bacteriocin activity of the concentrated supernatant was then tested by using agar well diffusion assay [36]. The indicator strain *L. monocytogenes* was grown for overnight and spread onto the BHI agar plate. Wells were made on the plate with sterile cork borer (7 mm in diameter, MRS Scientific Ltd., Wickford, UK), and the bottom of well was sealed with 20 μl of agar. After the concentrated supernatant was loaded, plates were incubated overnight at 37 °C.

### Identification of Leucocin C Using SDS-PAGE and Gel Overlay

Tricine-SDS-PAGE (16.5% resolving gel, 10% spacer gel) [37] and gel overlay assay [38] were performed to verify the presence of leucocin C in the supernatant. For SDS-PAGE, proteins from 100 μl of concentrated supernatant were collected with 10 μl of StrataClean resin (Agilent Technologies, Santa Clara, CA, USA). The resin beads were resuspended with 40 μl of Tricine-SDS-PAGE loading buffer (Bio-Rad, Hercules, CA, USA) to release the proteins. The mixture was boiled for 3 min and insoluble fraction was removed by centrifugation (10,000×*g*, 1 min). The soluble proteins were then analyzed by Tricine-SDS-PAGE. Two identical gels were prepared under the same condition. Ten microliters of concentrated Sb-LecC supernatant was loaded into the gel, and the electrophoresis was performed at 4 °C under 30 V for 30 min and 200 V for the rest of 2 h. After electrophoresis, SDS-PAGE gels were fixed for 30 min in the fixing solution (50% methanol, 10% acetic acid, 100 mM ammonium acetate). Then, one gel was stained with Coomassie Brilliant Blue R-250 (Sigma-Aldrich). The other gel was rinsed in distilled water for 2 h, then placed on BHI agar plate, and covered with 15 ml BHI soft agar containing 200 μl of the overnight culture of *L. monocytogenes*. The plate with gel was incubated overnight at 37 °C.

### Growth Profile

Bioscreen C™ Automated Microbiology Growth Curve Analysis System (Growth Curves, Helsinki, Finland) was used to determine the growth profile of yeasts. A single colony was inoculated into YPD medium and grown for overnight. On the next day, the fresh overnight culture was transferred to pre-warmed YPD medium with 2% inoculum. Two hundred microliters of the mixture were loaded into the honeycomb plate. The Bioscreen C™ system collected the optical density at 600 nm (OD_600_) every 1 h at 37 °C with continuous shaking. To measure the growth profile of yeasts under various pH conditions, YPD media were adjusted to pH 2 or 4, respectively.

### *L. monocytogenes* Killing Assay Using Recombinant S*. boulardii* Cells

In order to further quantify the capacity of Sb-LecC cells against *L. monocytogenes*, we tested the antimicrobial activity of the cell cultures without antibiotic selection pressure. First, Sb-LecC and *L. monocytogenes* were separately grown overnight. Colony forming unit (CFU) was calculated to obtain the initial cell numbers of *L. monocytogenes*. Then, *L. monocytogenes* was serially diluted (up to 1:1000) with Ringer’s solution. Sb-LecC cells were centrifuged and washed two times with Ringer’s solution to remove the antibiotic blasticidin S. After final resuspension in YPD medium, 900 μl of Sb-LecC cells were mixed with 100 μl of different concentrations of *L. monocytogenes*. We used two experimental approaches: (i) yeasts and *Listeria* cells were incubated in YPD medium with shaking, and (ii) mixed cells were centrifuged (5000×*g*, 3 min) to form a pellet, thereby reducing the physical distance between the cells. After incubation at 37 °C for 4 h, the viable cells of *L. monocytogenes* were determined by colony counting after serial dilutions. Nystatin of 50 μg/ml (Sigma-Aldrich) [39] was added into BHI plates to kill yeast cells for counting *Listeria* colonies. *Listeria* incubated without yeast and *Listeria* incubated with the yeast Sb-vector were used as controls. Then, the survival ratio was calculated based on the ratio between viable cell numbers and initial cell numbers of *Listeria*. If the survival ratio was less than one, we would consider that Sb-LecC killed *Listeria*. Survival ratio higher than one indicates that *Listeria* had grown.

## Results

### Cloning of the Leucocin C Gene *lecC* in *S. boulardii*

The aim of this work was to improve the probiotic potential of *S. boulardii* CNCM I-745 by enabling it to secrete the antimicrobial peptide leucocin C from *Leuconostoc carnosum*. Our strategy for this was to clone the leucocin C gene *lecC* with a yeast secretion signal in a plasmid vector. The *lecC* expression plasmid and the empty vector were then introduced into *S. boulardii*, resulting in the strains Sb-LecC and Sb-vector. The presence of the plasmids in *S. boulardii* was confirmed by PCR using plasmid specific primers. Correct bands of 1400 and 1050 bp were obtained from Sb-LecC and Sb-vector, respectively (Online Source 1). No band was obtained from the *S. boulardii* wild-type strain.

### Antimicrobial Activity of *S. boulardii* Sb-LecC

Antimicrobial activity of the constructed strain *S. boulardii* Sb-LecC was tested against *L. monocytogenes*. In the agar inhibition assay, Sb-LecC cells caused a clear inhibition zone on *Listeria* lawn, whereas the Sb*-*wild type and Sb-vector did not inhibit *Listeria* (Online Source 2a). To examine the secretion of the antimicrobial substance, the strains were cultured in liquid media. Concentrated supernatant of Sb-LecC and leucocin C from *L. lactis* NZ9000 as a control showed inhibition against *L. monocytogenes*, whereas the supernatants from Sb-wild type and Sb-vector were not antimicrobial (Online Source 2b). These results demonstrated the secretion of antilisterial compound by the Sb-LecC strain.

### Identifying Leucocin C in Sb-LecC Growth Supernatant

To verify that the inhibition effect of the strain Sb-LecC was caused by secreted leucocin C, concentrated cell-free supernatant of the Sb-LecC was analyzed in Coomassie-stained SDS-PAGE and in gel overlay assay. In stained SDS-gel, concentrated supernatant of Sb-LecC gave a band of the same size as leucocin C from *L. lactis*, between 4.6 and 10 kDa (Online Source 3a). According to the amino acid sequence, the calculated molecular weight of leucocin C is 4.6 kDa. To confirm that the obtained band was the active antimicrobial leucocin C, the supernatants were loaded into another SDS-gel, which was subjected to overlay assay with *L. monocytogenes*. Sb-LecC supernatant and leucocin C from *L. lactis* formed inhibition zones corresponding to the bands in the stained SDS-gel (Online Source 3b). Neither the supernatant of Sb-wild type nor the supernatant of Sb-vector showed any inhibition. Therefore, it could be concluded that the constructed Sb-LecC secretes leucocin C.

### Growth of *S. boulardii* Strains

**Fig. 2:**
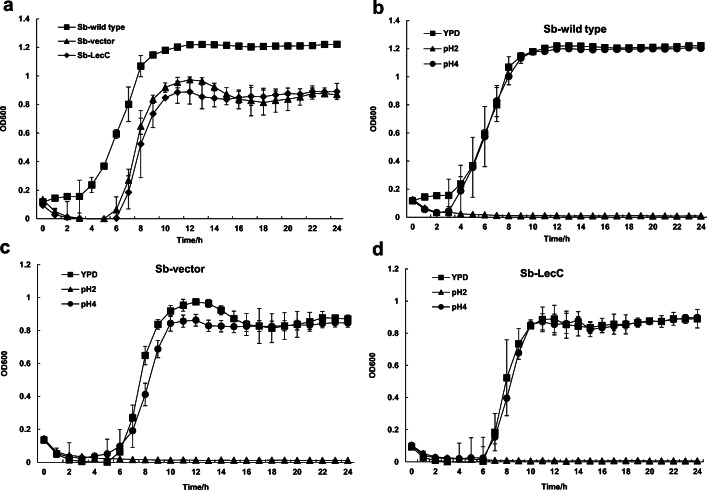
Growth of *S. boulardii* strains. **a** Growth curve of Sb*-*wild type, Sb-vector, and Sb-LecC in YPD medium. **b**–**d** Growth curves of Sb*-*wild type, Sb-vector, and Sb-LecC in YPD medium of pH 2 and pH 4. All experiments were performed in triplicate. Data is presented as mean value with standard deviation

The effect of the introduced plasmids on growth was investigated. Carrying heterologous plasmid made the lag phase longer and decreased the cell concentration in the stationary growth phase of both Sb-LecC and Sb-vector strains, compared with the wild-type strain (Fig. 2a). However, as there was no significant difference between the growth of Sb-LecC and Sb-vector strain, it can be deduced that the production of the bacteriocin did not disturb the growth.

In order to determine whether the transformants could tolerate acidity similar to that in the human stomach, the growth of *S. boulardii* strains was tested at pH 2 and pH 4. All three *S. boulardii* strains grew in the same way at pH 4 as in YPD medium without pH adjustment, but they could not grow at pH 2 (Fig. 2b–d). Nevertheless, all the strains were still viable after 24-h incubation at pH 2 (data not shown). These results indicated that even though the growth of Sb-LecC and Sb-vector was disturbed by introduced plasmids, their resistance to low pH was maintained.

### Killing of *L. monocytogenes* Without Selection Pressure

**Fig. 3:**
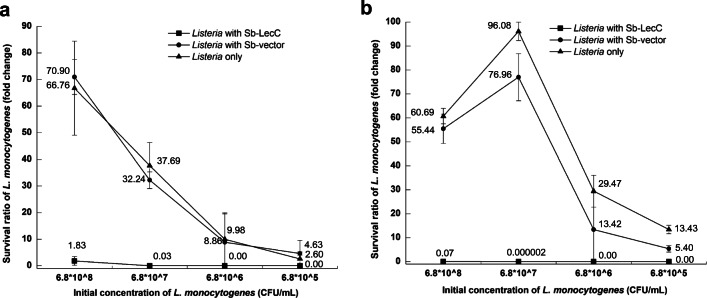
Killing of *Listeria* by *S. boulardii*. **a** *S. boulardii* and *L. monocytogenes* were co-cultured in the YPD medium with shaking. **b** *S. boulardii* and *L. monocytogenes* were incubated in the form of cell pellet after centrifugation to make a closer contact. The survival ratio of *L. monocytogenes* after incubation is shown in charts. The cell density of original *L. monocytogenes* culture was 6.8 × 10^9^ CFU/ml, and then it was diluted to 6.8 × 10^8^–6.8 × 10^5^ CFU/ml for incubation. The cell density of *S. boulardii* added was 3.4 × 10^7^ CFU/ml. All experiments were performed in triplicate. All data is presented as mean value with standard deviation

The last aim in this study was to assess the killing capacity of Sb-LecC, as well as to determine whether the strain can retain its antimicrobial activity without selection pressure. To answer these questions, *S. boulardii* strains were co-cultured with serially diluted *L. monocytogenes* in media without blasticidin S. As shown in Fig. 3a, *S. boulardii* Sb-LecC killed *Listeria* effectively. When *L. monocytogenes* was incubated with Sb-LecC at the initial *Listeria* concentration of 6.8 × 10^7^ CFU/ml, only 3% of *Listeria* cells were viable. With lower initial *Listeria* concentrations, no live cells were found. Although *Listeria* count in liquid culture with Sb-LecC almost doubled (1.83) at the initial cell concentration of 6.8 × 10^8^ CFU/ml, its survival ratio was much lower than when incubated with Sb-vector (70.90) or without yeast (66.76).

In addition, we wanted to examine if the killing efficiency could be increased when the bacteriocin producing yeast and *Listeria* were closer to each other. To test that, *S. boulardii* and *Listeria* cultures were mixed, the mixtures were centrifuged, supernatants were removed, and the cell pellets were incubated per se to keep the cells in close contact. Compared with the results from liquid co-cultures, Sb-LecC killed *L. monocytogenes* more effectively when the two strains were incubated in pellet, also at the highest initial listerial cell concentration (Fig. 3b). The Sb-LecC strain killed 93% of *Listeria* cells at the highest initial concentration of 6.8 × 10^8^ CFU/ml. At the initial concentration of 6.8 × 10^7^ CFU/ml, the amount of viable *Listeria* dropped after incubating with *S. boulardii* Sb-LecC, with only a marginal survival ratio of 2 × 10^−6^. With lower initial concentrations, no live *L. monocytogenes* cells were found (Fig. 3b). Quite the opposite, when *Listeria* was incubated without yeast cells, or with the *S. boulardii* vector strain, *Listeria* counts increased after the incubation. These results indicated that *S. boulardii* producing leucocin C killed the pathogen *L. monocytogenes* without antibiotic selection pressure and that the killing efficiency was higher when the cells were in close proximity.

## Discussion

In this study, the probiotic yeast *S. boulardii* CNCM I-745 was used as the host to secrete antilisterial peptide leucocin C. Some of the unique features of *S. boulardii* make it a superior drug delivery vehicle compared with other microorganisms. For example, *S. boulardii* exhibits better heat and acid tolerance than *S. cerevisiae* [1], making it a stronger survivor under the harsh conditions in the gastrointestinal tract. In addition, since *S. boulardii* is not a natural colonizer in the intestine [1, 40], it can easily be removed after treatment [41]. Moreover, nowadays yeast cultures can be produced in large scale at low cost, which makes the genetically modified *S. boulardii* an economical vehicle for drug delivery.

To realize the purposes mentioned above, it is essential to examine or develop tools for genetic manipulation for *S. boulardii*. To date, the genome sequences of a few *S. boulardii* strains, such as ATCC MYA-796 [42], Unique 28, and CNCM I-745 [43], have been published, but the information about promoters or functional genes of *S. boulardii* is still scarce. One of the few studies about *S. boulardii* genetics is the work by Douradinha et al. [9], who confirmed the existence of the constitutive promoters *PGK1*, *PYK1*, and *ENO1* in the genome of the strain ATCC MYA-796. Therefore, genetic manipulation of *S. boulardii* mainly relies on the genetic tools for *S. cerevisiae*. For example, *S. cerevisiae* promoters like *TEF1* and *PGK1* [12], *TDH3* [14], and *Gal1* [15] were successfully applied for the heterologous protein expression in *S. boulardii*. In our study, expression of *lecC* was mediated by the constitutive promoter *TEF1*, which has been shown to be constantly active during cultivation [29, 30]. To facilitate the secretion of leucocin C, the commonly used α-mating factor signal sequence [31] was fused to the *lecC* gene. As for the selection marker, we have previously tested the sensitivity of wild-type *S. boulardii* to blasticidin S and determined its minimum lethal dose (20 μg/ml) [28]. The successful secretion of leucocin C in our study demonstrated that the *TEF1* promoter and α-mating factor signal sequence of *S. cerevisiae* can be used for *S. boulardii* CNCM I-745. Yet, the vector used here is a high copy number plasmid, which decreased the growth of the host [44]. Indeed, the transformed strains Sb-vector and Sb-LecC exhibited a prolonged lag phase and yielded a lower cell density at the stationary phase (Fig. 2a) compared with the Sb-wild type strain, even without the selective agent blasticidin S in the medium (data not shown).

In SDS-PAGE and gel overlay assay, the leucocin C secreted by Sb-LecC had a corresponding molecular weight and inhibition band between 4.6 and 10 kDa. These results are in consistence with the results of Fu et al. [45] and Wan et al. [27], which both used *L. lactis* as the host*.* SDS-PAGE gives only a rough estimation of the molecular weight [46], explaining inaccurate results when molecular weight of leucocin C was determined with SDS-PAGE. However, our aim here was only to demonstrate the presence and activity of leucocin C in the Sb-LecC supernatant, as when we tested its antimicrobial activity against *L. monocytogenes*, it indeed gave an inhibition halo (Online Source 2). These results revealed that *S. boulardii* is able to act as a host to secrete heterologous and active leucocin C.

Undoubtedly, it is important that the yeast to be used as a probiotic survives and, if possible, is metabolically active in the gastrointestinal tract. In previous studies, *S. boulardii* has shown an enhanced growth compared with *S. cerevisiae* in media adjusted to pH 4 [1, 47]. Thus, we investigated whether the transformants we constructed could retain tolerance to low pH, resembling the condition in stomach. The Sb-LecC constructed in this study grew well at pH 4 (Fig. 2d), and it could recover after 24-h incubation at pH 2, indicating that it survives the acidic conditions in human stomach and can possibly regain its activity in human intestinal environment. Thus, it seems possible that the leucocin C-secreting *S. boulardii* we constructed can function as a vehicle to deliver leucocin C into the small intestine. Correspondingly, *S. boulardii* may be modified to secrete other recombinant therapeutics into human intestine. Michael et al. [10] constructed an IL-10 producing *S. boulardii* and tested its anti-inflammatory function in the colitis mice model. No significant differences were observed between the treated and untreated mice groups, suggesting that IL-10 was not secreted in the intestine in sufficient quantities to decrease the inflammatory response. A later research by Hudson et al. [13] demonstrated that their GFP-producing recombinant *S. boulardii* strain could be recovered from Peyer’s patches of mice fed with the strain and that the isolated yeast cells had maintained their GFP production capacity. Most recently, Bagherpour et al. [15] successfully applied an OVA-producing *S. boulardii* to bring antigenic peptide ovalbumin into intestinal lumen via mice oral administration and observed a significant increase of antibody response in treated group compared with control groups. This work indicated the potential of this new delivery platform.

The use of probiotic microorganisms expressing antimicrobial peptides has been studied as a dual therapy to control bacterial infectious diseases [48]. Probiotic bacteria like *Lactococcus lactis* [49], *Lactobacillus plantarum* [50], and *Propionibacterium freudenreichii* [51] have been successfully applied as hosts to produce bacteriocin. As probiotic yeast, *S. boulardii* has no direct antimicrobial effects, except binding to some enteric pathogens like *Salmonella* [52], *E. coli* [53], and *Shigella* [54]. In our case, we focused on improving the probiotic effects of *S. boulardii* by adding the ability to kill *Listeria*. Thus, the leucocin C expressed in this study enabled *S. boulardii* to directly kill *Listeria*. As a foodborne pathogen, *L. monocytogenes* can get into the human gastrointestinal tract, interacting with intestinal epithelium [55, 56], and cause gastroenteritis [57]. It is important to eliminate or prevent *L. monocytogenes* in the human intestine. In this study, we demonstrated that the Sb-LecC could efficiently kill *L. monocytogenes* in vitro. When we incubated *L. monocytogenes* and Sb-LecC cells in a pellet formed by centrifugation, *L. monocytogenes* was killed more efficiently compared with incubating free cells together in liquid culture. Clearly, incubating the cells in the centrifugation pellet provides a better chance for cell contact and interactions, and leucocin C could thereby easily reach the target. The concentration of leucocin C in the cell pellet is likely to be higher than when leucocin C is freely secreted to the growth medium, which is the case when the two strains are co-cultured in a liquid culture. The results also demonstrated that the leucocin C secretion was relatively stable, as Sb-LecC was still secreting active leucocin C even without selection pressure, and the yield was enough to kill *Listeria*. Washing Sb-LecC cells directly after they were separated from the growth medium did not release leucocin C into the supernatant (data not shown), suggesting that produced leucocin C does not stick to the outer surface of Sb-LecC cells.

Taken together, our study showed that it is possible to add antibacterial capacity to the probiotic yeast *S. boulardii* by making it to secrete bacteriocin. In addition, we gave an insight about the possibility of applying genetically engineered *S. boulardii* as a carrier for therapeutics delivery. Further studies will focus on integrating the expression cassette into the chromosome of the *S. boulardii* and future testing of their improved probiotic effects in vivo in animal model.

## Electronic Supplementary Material

ESM 1 (DOCX 487 kb)

ESM 2 (DOCX 24111 kb)

ESM 3 (DOCX 2398 kb)
