# Supplementary material for: Heterologous Expression of the Leuconostoc Bacteriocin Leucocin C in Probiotic Yeast Saccharomyces boulardii
Source: Probiotics Antimicrob Proteins. 2020 Jun 21;13(1):229–37. doi: 10.1007/s12602-020-09676-1 (PMC7904741; doi:10.1007/s12602-020-09676-1)
Supplement: Supplementary file 1 — (DOCX 487 kb) [file 12602_2020_9676_MOESM1_ESM.docx]

**Heterologous expression of the *Leuconostoc* bacteriocin leucocin C in probiotic yeast *Saccharomyces boulardii***

**Probiotics and Antimicrobial Proteins**

Ran Li^a,^ *, Xing Wan^a^, Timo M. Takala^a^, Per E.J. Saris^a^

**Affiliation:**

Department of Microbiology, Faculty of Agriculture and Forestry, University of Helsinki^a^

***Corresponding author:** Ran Li, [ran.li@helsinki.fi](mailto:ran.li@helsinki.fi), telephone: +358 415860783


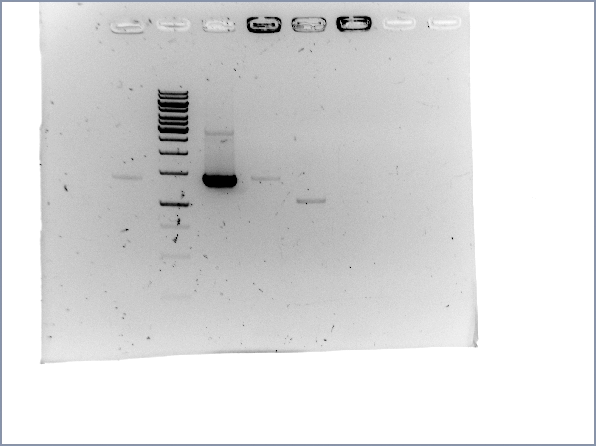


1500 bp

1000 bp

Marker

PC

Sb-LecC

Sb-vector

Sb-wild type

NC

**Online Source 1** PCR verification of the pSF-Blast-*lecC* plasmid in Sb-LecC strain. PC: positive control, synthesized pSF-Blast-*lecC* plasmid; Sb-LecC: cells of Sb-LecC as template; Sb-vector: cells of Sb-vector as template; Sb-wild type: cells of *S. boulardii* wild type as template; NC: negative control of the PCR reaction, sterilized water as the template. Plasmid specific primers were used, which yield a 1400-bp band from plasmid pSF-Blast-*lecC* and a 1050-bp from the empty vector. A 1400-bp PCR product is detected from Sb-LecC (black arrow)
