# Supplementary material for: Heterologous Expression of the Leuconostoc Bacteriocin Leucocin C in Probiotic Yeast Saccharomyces boulardii
Source: Probiotics Antimicrob Proteins. 2020 Jun 21;13(1):229–37. doi: 10.1007/s12602-020-09676-1 (PMC7904741; doi:10.1007/s12602-020-09676-1)
Supplement: Supplementary file 2 — (DOCX 24111 kb) [file 12602_2020_9676_MOESM2_ESM.docx]

**Heterologous expression of the *Leuconostoc* bacteriocin leucocin C in probiotic yeast *Saccharomyces boulardii***

**Probiotics and Antimicrobial Proteins**

Ran Li^a,^ *, Xing Wan^a^, Timo M. Takala^a^, Per E.J. Saris^a^

**Affiliation:**

Department of Microbiology, Faculty of Agriculture and Forestry, University of Helsinki^a^

***Corresponding author:** Ran Li, [ran.li@helsinki.fi](mailto:ran.li@helsinki.fi), telephone: +358 415860783


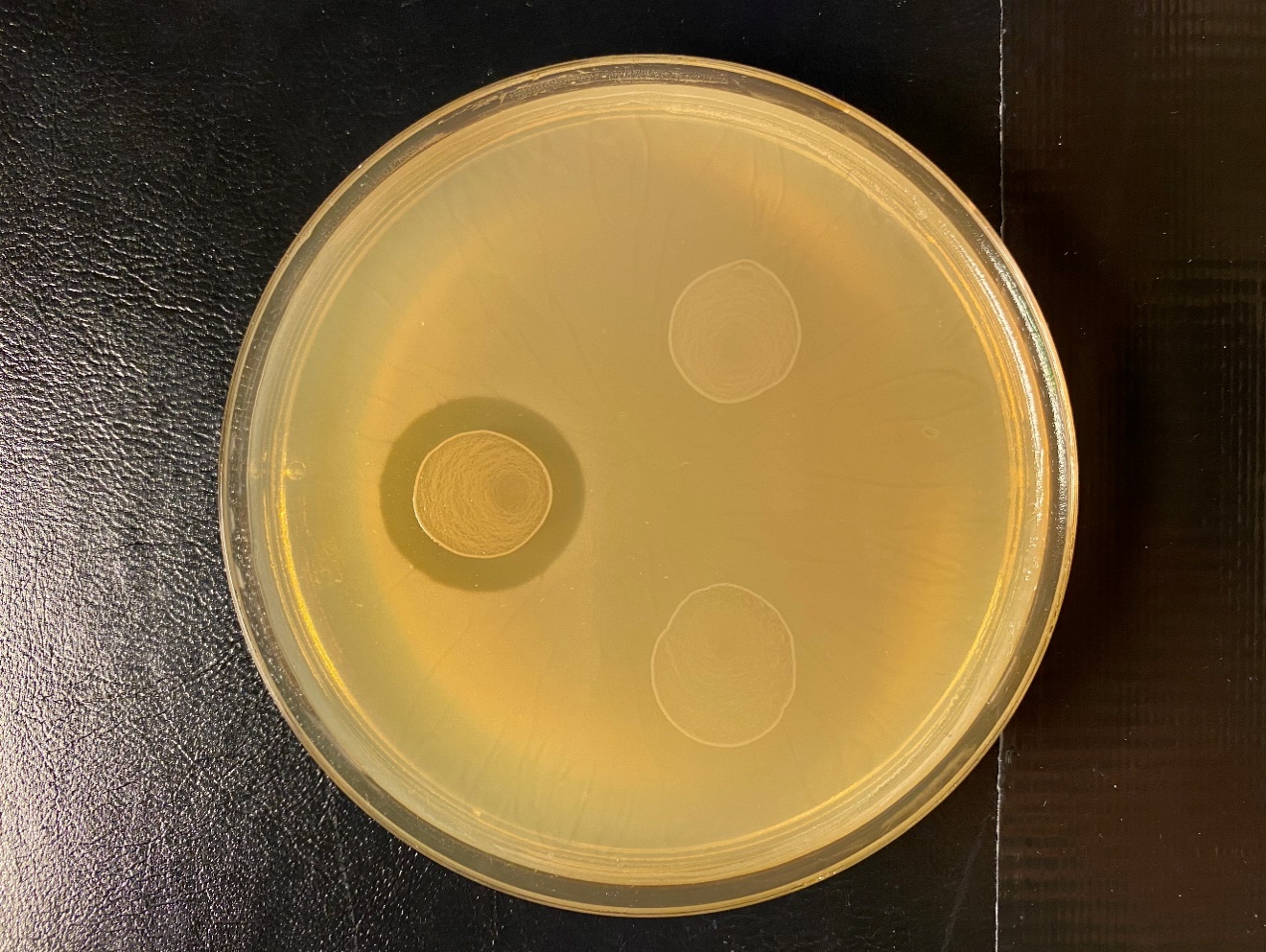

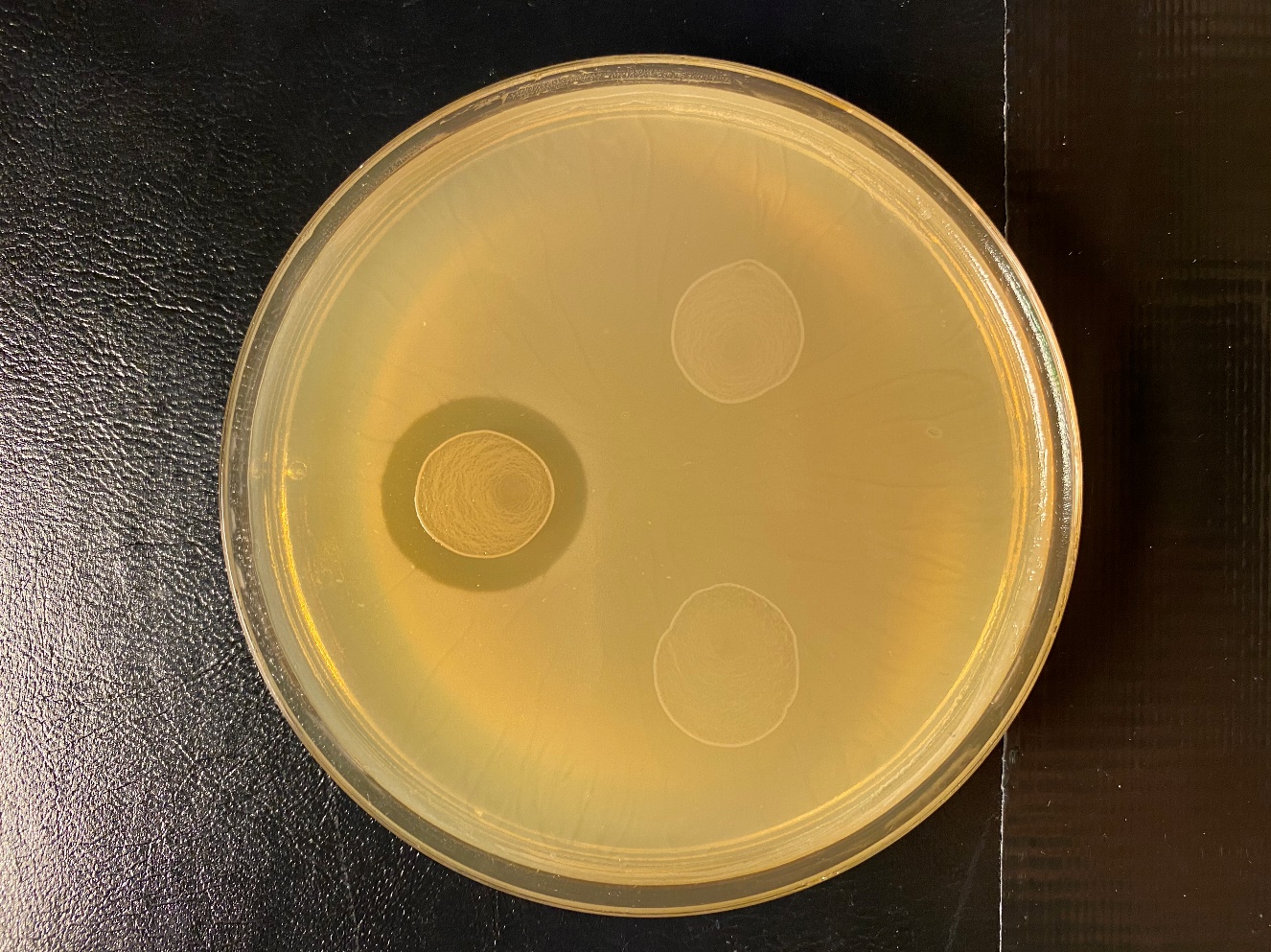

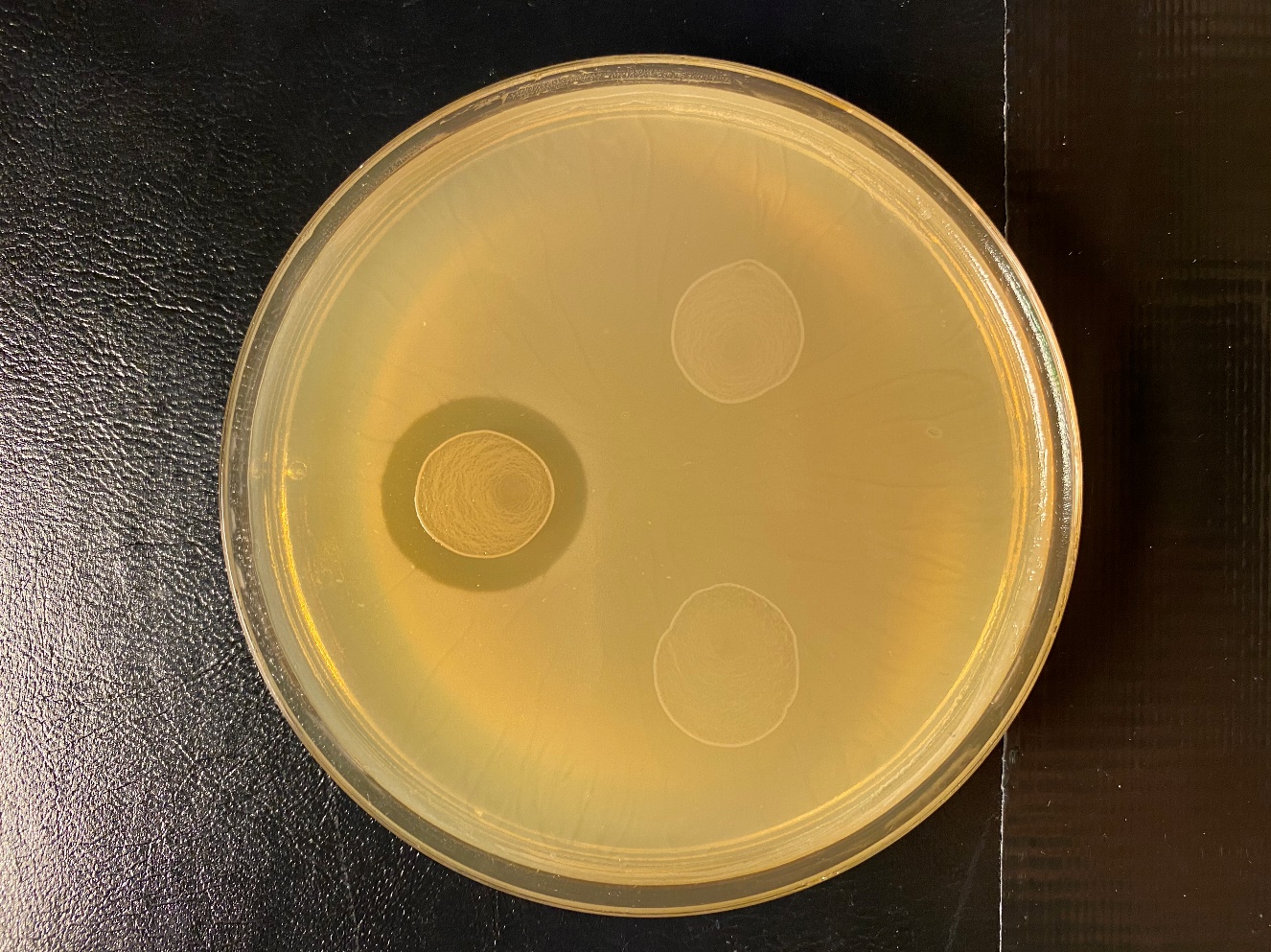


Sb-LecC Sb-vector Sb-wild type

**a**

Sb-wild type sup Sb-vector sup

PC Sb-LecC sup

**b**


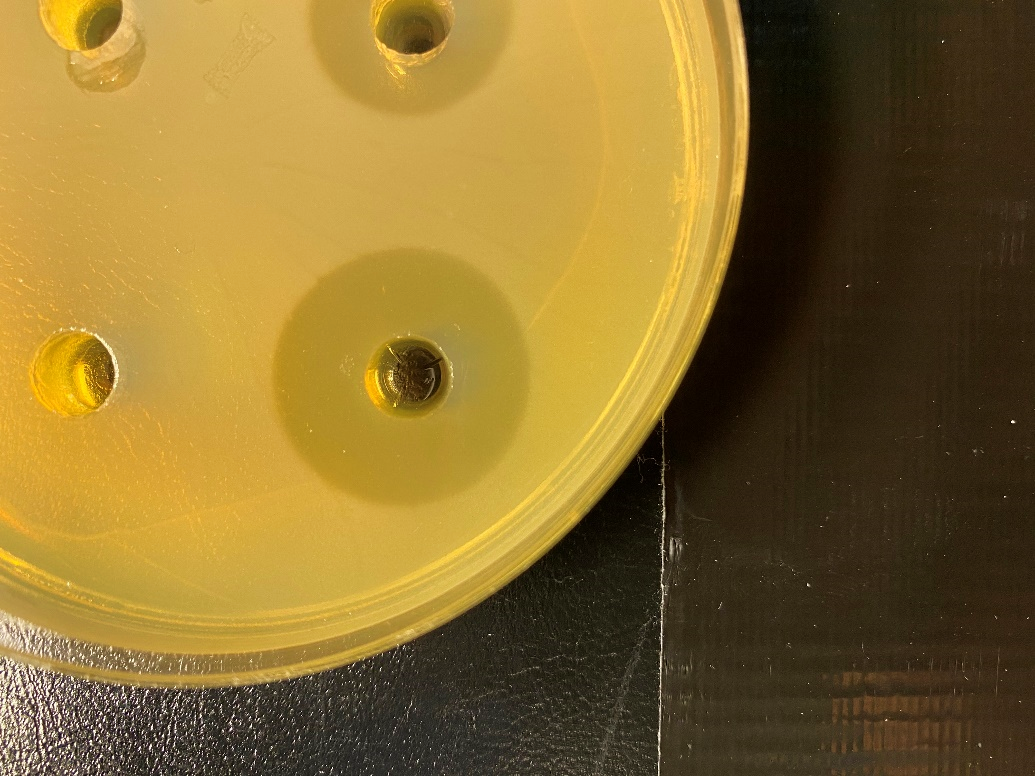

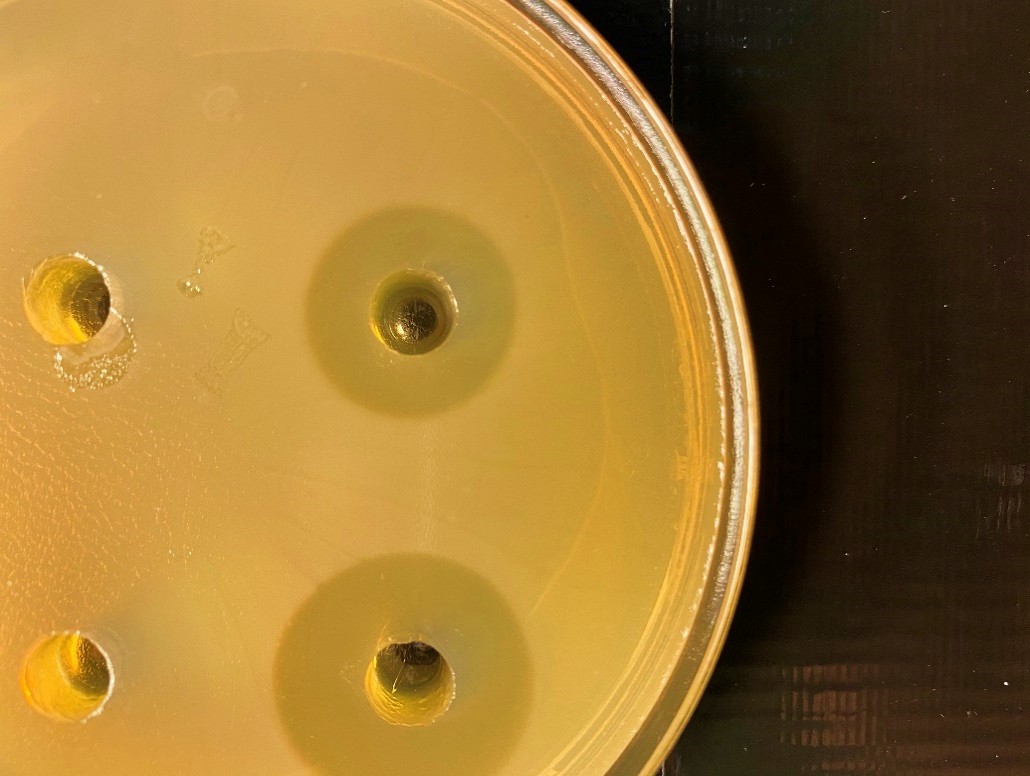

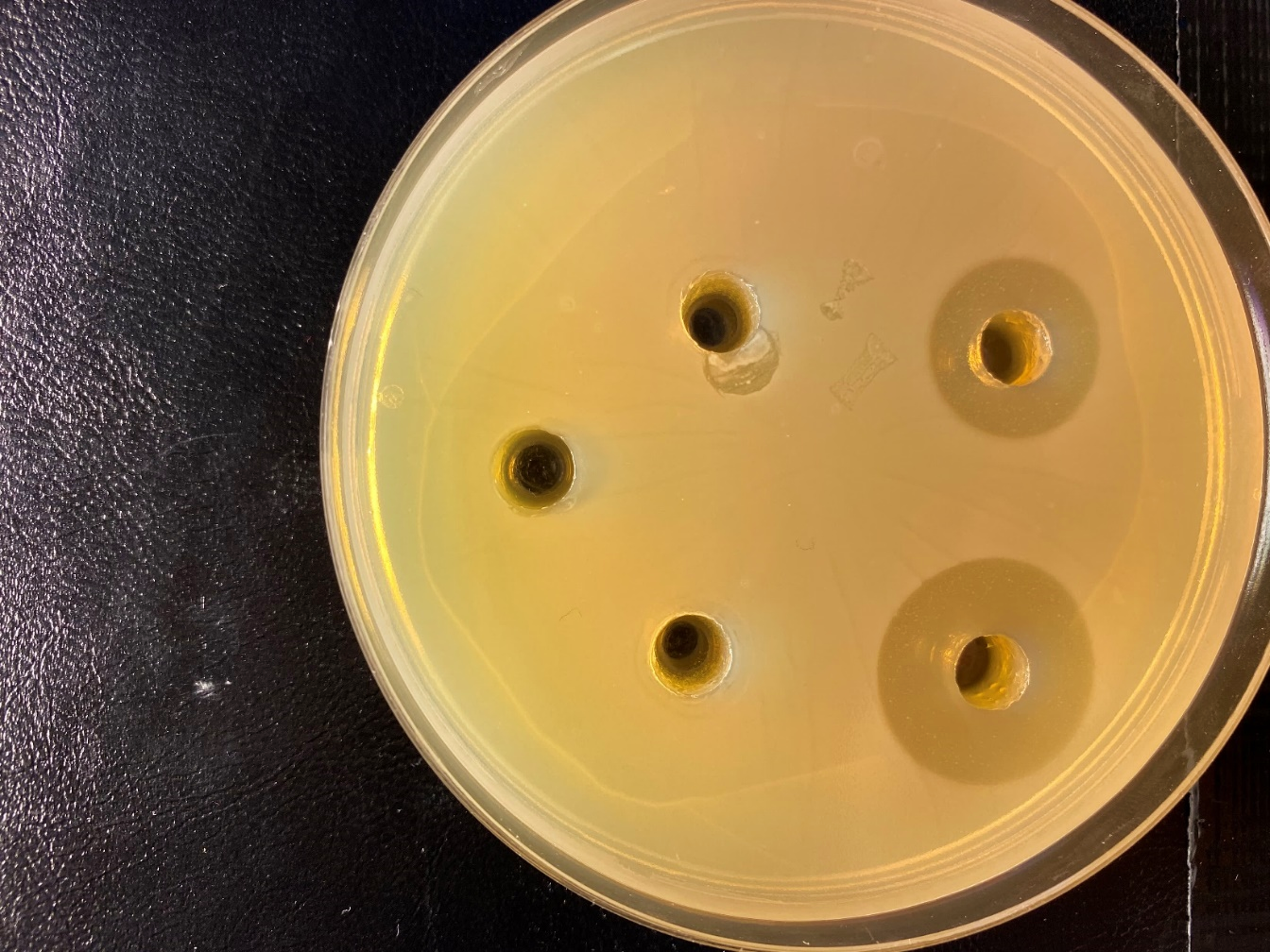

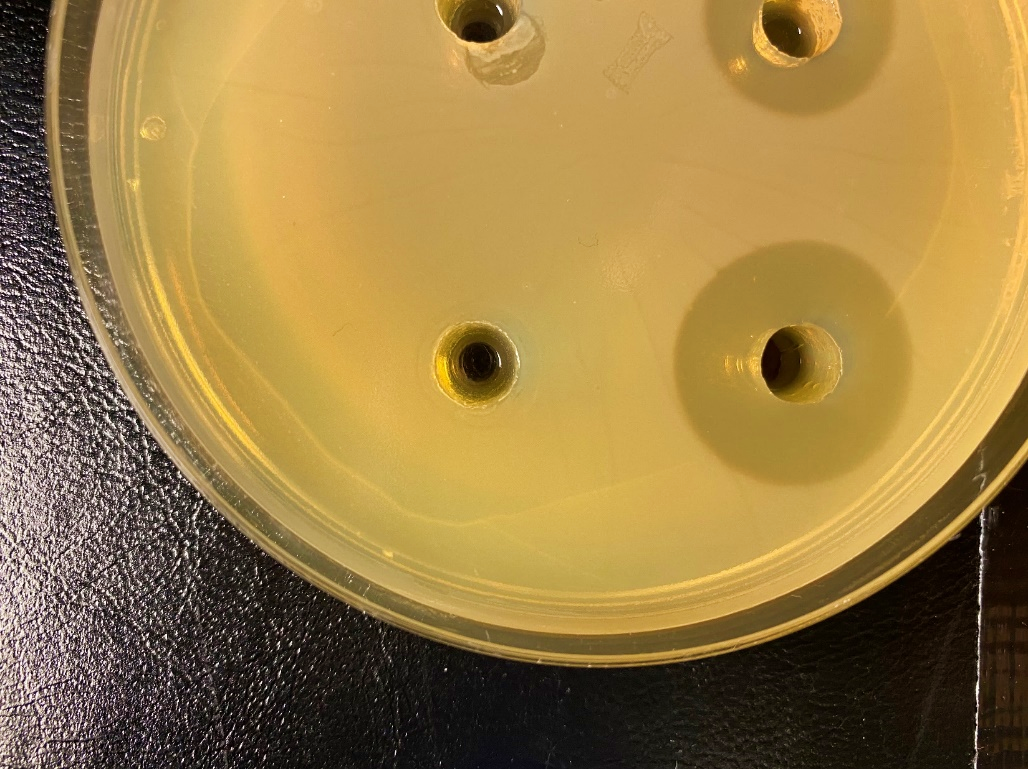


**Online Source 2** Antimicrobial tests. (a) Agar inhibition assay. Washed yeast cells were added on *Listeria* indicator plate. The cell density of Sb-LecC culture was 5.1×10^7^ CFU/ml, Sb-vector was 5.4×10^7^ CFU/ml, and Sb-wild type was 7.4×10^7^ CFU/ml. Inhibition halo is observed only in Sb-LecC. This indicates that Sb-LecC had produced substance, which inhibited the growth of *L. monocytogenes*. (b) Agar well diffusion assay. All the samples loaded were from concentrated supernatants. Sb-wild type sup: 90 μl of the supernatant from wild-type *S. boulardii*; Sb-vector sup: 90 μl of the supernatant from Sb-vector strain; PC: positive control, 10 μl of the supernatant from the leucocin C producing *L. lactis* NZ9000 strain; Sb-LecC sup: 90 μl of the supernatant from Sb-LecC
