# Supplementary material for: Heterologous Expression of the Leuconostoc Bacteriocin Leucocin C in Probiotic Yeast Saccharomyces boulardii
Source: Probiotics Antimicrob Proteins. 2020 Jun 21;13(1):229–37. doi: 10.1007/s12602-020-09676-1 (PMC7904741; doi:10.1007/s12602-020-09676-1)
Supplement: Supplementary file 3 — (DOCX 2398 kb) [file 12602_2020_9676_MOESM3_ESM.docx]

**Heterologous expression of the *Leuconostoc* bacteriocin leucocin C in probiotic yeast *Saccharomyces boulardii***

**Probiotics and Antimicrobial Proteins**

Ran Li^a,^ *, Xing Wan^a^, Timo M. Takala^a^, Per E.J. Saris^a^

**Affiliation:**

Department of Microbiology, Faculty of Agriculture and Forestry, University of Helsinki^a^

***Corresponding author:** Ran Li, [ran.li@helsinki.fi](mailto:ran.li@helsinki.fi), telephone: +358 415860783

PC

**a**

40

25

15

10

4.6

kDa

Sb-LecC sup

Marker

**b**

PC

Marker

Sb-vector sup

Sb-wild type sup

Sb-LecC sup

40

25

15

4.6

10

kDa


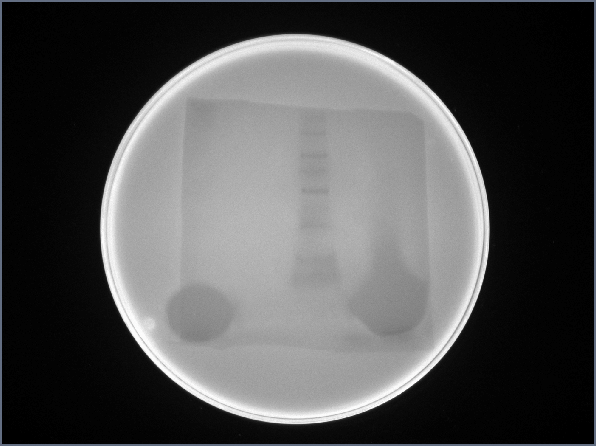


**Online Source 3** Identification of leucocin C in the concentrated supernatant of Sb-LecC. (a) Tricine-SDS-PAGE (16.5% resolving gel, 10% spacer gel), Coomassie blue stained gel. (b) Gel overlay assay: gel on BHI agar covered with BHI soft agar containing *L. monocytogenes*. Putative leucocin C in Sb-LecC concentrated supernatant appears between 4.6 and10 kDa as a band in (a; black arrow) and a corresponding inhibition zone in (b), which is in consistence with the positive control (leucocin C from *L. lactis* NZ9000, white arrow). Negative controls (Sb-vector supernatant and Sb-wild type supernatant) do not show any inhibition zones
